# Heterogeneity of borderline personality disorder symptoms in help-seeking adolescents

**DOI:** 10.1186/s40479-021-00147-9

**Published:** 2021-02-26

**Authors:** Marialuisa Cavelti, Stefan Lerch, Denisa Ghinea, Gloria Fischer-Waldschmidt, Franz Resch, Julian Koenig, Michael Kaess

**Affiliations:** 1 University Hospital for Child and Adolescent Psychiatry and Psychotherapy, University of Bern, Bolligenstrasse 111, 3000, Bern 60, Switzerland; 2 Section for Translational Psychobiology in Child and Adolescent Psychiatry, Department of Child and Adolescent Psychiatry, Centre for Psychosocial Medicine, University of Heidelberg, Heidelberg, Germany; 3 Clinic for Child and Adolescent Psychiatry, Centre for Psychosocial Medicine, University of Heidelberg, Heidelberg, Germany; 4 Section for Experimental Child and Adolescent Psychiatry, Department of Child and Adolescent Psychiatry, Centre for Psychosocial Medicine, University of Heidelberg, Heidelberg, Germany

**Keywords:** Adolescence, Borderline personality disorder, Emotionally unstable personality disorder, Categorical and dimensional models of personality, Factor mixture models

## Abstract

**Background:**

The heterogeneous presentation of borderline personality disorder (BPD) represents a clinical challenge. There is an ongoing scientific debate whether the heterogeneity can best be understood in terms of qualitative (categorical) or quantitative (dimensional) differences between individuals. The present study examined the latent structure of BPD in adolescents.

**Methods:**

Five-hundred and six outpatients aged 12 to 17 years with risk-taking and/or self-harming behavior were assessed at baseline and one-year follow-up. Latent class analysis (corresponding with the categorical approach), factor analysis (corresponding with the dimensional approach), and factor mixture models (allowing for both categorical and dimensional aspects) were applied to the DSM-IV BPD criteria.

**Results:**

The best fitting model distinguished between a majority class with high probabilities for all BPD criteria (“borderline group”) and a minority class with high probabilities for the impulsivity and anger criteria only (“impulsive group”). Sex significantly affected latent class membership, and both a latent factor and age explained within-class variability. The borderline group primarily consisted of females, frequently reported adverse childhood experiences, scored high on the emotion dysregulation and inhibitedness personality traits, and was associated with internalizing psychopathology. In contrast, the impulsive group primarily consisted of males, scored high on the dissocial behavior personality trait, and was associated with externalizing psychopathology. After one year, the two groups showed similar clinical improvement.

**Conclusions:**

The study provides evidence for two distinct subgroups of adolescents with BPD features that resemble the subtypes of the ICD-10 emotionally unstable personality disorder. More research is needed to further investigate the diagnostic stability of the two groups over time and potential differential treatment indications.

**Supplementary Information:**

The online version contains supplementary material available at 10.1186/s40479-021-00147-9.

## Introduction

Borderline personality disorder (BPD) is a severe mental disorder that is characterized by interpersonal instability, cognitive and self-disturbance, and affective and behavioral dysregulation [1]. It usually emerges during adolescence and early adulthood, and can interfere with key developmental tasks in this period of life [2]. In the long-term, individuals with BPD show psychosocial impairments that are more severe and enduring than many other major psychiatric disorders [3–5]. Accordingly, early detection and intervention for BPD has become a novel public health priority that aims at preventing adverse personal, social, and economic consequences of BPD [6]. Today, there is a broad evidence-based consensus that BPD is a valid and reliable diagnosis in adolescence, with prevalence rates ranging from 1 to 3% in the general population to 30–50% in inpatients [7]. In addition, several studies suggest that early intervention, including indicated prevention for those with precursors or early features of BPD (sub-threshold disorder) and treatment for those with first presentation, full-threshold BPD, is feasible and effective [8, 9].

The clinical presentation of BPD can greatly vary between individuals [10] as well as within individuals over time [4]. Concerning inter-individual variability, the Diagnostic and Statistical Manual of Mental Disorders, 5th Edition (DSM-5), lists in Section II nine criteria for BPD of which at least five have to be met for the diagnosis [11]. This results in 256 possible combinations that can lead to the diagnosis. Two patients diagnosed with BPD may not overlap in more than one criterion. The International Statistical Classification of Diseases and Related Health Problems, 10th Revision, (ICD-10) (World Health Organization, 1992) addresses the issue of significant inter-individual variability by proposing two subtypes of the emotionally unstable personality disorder: an impulsive type (F60.30) characterized by emotional instability (outbursts of angry or threatening behavior) and impulsivity only, and a borderline type (F60.31), which additionally features interpersonal issues, identity disturbance, self-destructive behavior, and chronic feelings of emptiness. However, only the borderline type will remain in the 11th revision of the ICD [12]. There is initial evidence indicating that inter-individual variability in BPD presentation may be partly explained by gender, even though results are inconsistent [13, 14]. Concerning within-individual variability, evidence indicates that “acute” symptoms such as impulsivity, self-harm and anger dominate during adolescence, while more “chronic” symptoms such as interpersonal difficulties and feelings of emptiness come to the fore during adulthood [15]. The phenomenological heterogeneity of the disorder represents a major challenge, both for clinical practice and research. It can impede efforts to clarify the etiology of the disorder, complicate diagnosis, and challenge disorder-specific treatments.

There is an ongoing scientific debate whether the phenomenological heterogeneity of BPD can be best understood in terms of qualitative (categorical) or quantitative (dimensional) differences between individuals. Conventionally, two analytical approaches have been applied to parse the phenomenological heterogeneity of BPD. The first is a person-centered approach that uses *Latent Class Analysis (LCA)* to classify individuals according to patterns of BPD criteria into subtypes that are thought to be homogeneous subgroups of the disorder. Any two randomly selected individuals are thought to are either the same or different depending on whether or not they stem from the same latent class [16, 17]. To date, LCA has been applied to DSM BPD criteria assessed in community and clinical samples, all but one [18] consisted of adults [19–23]. Most authors concluded that the classes reflect discrete points along a latent continuum of BPD severity [18–20, 22]. Two studies stand out from the others by reporting an “impulsive class”, including patients who only endorsed impulsivity (criterion 4) and inappropriate anger (criterion 8) at high rates, along with two or three classes with increasing BPD severity [21, 23]. Notably, these “impulsive classes” [21, 23] differed in the BPD criteria combination from “no/low severity classes” identified in other studies [18–20, 22].

The second analytical approach is variable-centered and uses *Factor Analysis (FA)* to reduce diagnostic criteria to a few underlying dimensions (latent factors). Individuals are thought to differ from each other according to their scores on the underlying latent factor(s) [16, 17]. To date, numerous studies have applied FA to the DSM BPD criteria in both adult and youth samples. Recently, Michonski et al. [10] reviewed the literature and concluded that across both adult and adolescent samples, there is more support for a single-factor solution than for any other factor model. In addition to the one-factor solution, another model that has been replicated within both adult and youth samples is Sanislow et al.’s [24] three-factor model. Notably, in Sanislow et al. [24]‘s model the three factors were highly correlated (.90–.99), suggesting that a one-factor structure underlying BPD criteria may be a more parsimonious solution [10].

*Factor Mixture Models (FMM)* are a new statistical advancement that incorporate both LCA and FA, thereby taking into account that the “true” nature of a latent construct such as BPD may include both categorical and dimensional aspects. FMM allow for the classification of individuals into subgroups and, at the same time, account for within-class heterogeneity by one or more latent factors [25]. There is a range of FMM variants that differ in their amount of measurement invariance and their interpretation [26]. Measurement invariance assesses the equivalence of latent factors across latent classes [27], and can take many forms depending on the parameters (i.e., factor means, factor covariances, item thresholds, factor loadings) that are specified as class-specific (see Table 1 in Supplementary Material (SM) for an overview about FMM variants according to Clark et al. [26]). To date, only two studies have applied FMM to probe the latent structure of BPD. Conway et al. [28] investigated a community sample of *N* = 700 adults at risk for psychopathology due to elevated rates of maternal depression, and reported that a FA model suggesting a single latent continuum of BPD pathology provided a better fit compared with LCA and FMM models. In contrast, the study by Hallquist and Pilkonis [29] found that in a mixed clinical and nonclinical sample of *N* = 362 adults, a FMM consisting of a symptomatic and asymptomatic latent class and a single factor representing severity outperformed LCA and FA models. A methodological reason for the diverging results may be that the two studies tested different FMM variants, with Conway et al. [28] fitting a stricter FMM variant than Hallquist and Pilkonis [29]. It is precisely the investigation of different model variants that makes FMM a particularly flexible tool to investigate the latent structure of psychological constructs. Its potential has not yet been fully explored with regard to the latent structure of BPD. In addition, no study to date has used FMM to probe the latent structure of BPD in adolescence.

**Table 1:** Primary diagnoses in the sample (*N* = 506)

ICD-10 diagnoses	Number of diagnoses within the sample	Percentage of patients with a diagnosis
Organic, including symptomatic, mental disorders (F00-F09)	0	0
Mental and behavioral disorders due to psychoactive substance use (F10-F19)	129	18.77
Schizophrenia, schizotypal and delusional disorders (F20-F29)	0	0
Affective disorders (F30-F39)	381	61.26
Neurotic, stress-related and somatoform disorders (F40-F49)	279	36.76
Behavioral syndromes associated with physiological disturbances and physical factors (F50-F59)	64	12.45
Disorders of adult personality and behavior (F60-F69)	245	40.12
Mental retardation (F70-F79)	0	0
Disorders of psychological development (F80-F89)	3	0.59
Behavioral and emotional disorders with onset in childhood and adolescence (F90-F99)	180	28.85

*Notes.* ICD-10 = International Statistical Classification of Diseases and Related Health Problems, 10th Revision*.* Multiple diagnoses per subject were possible

In order to address this research gap, we investigated the latent structure of DSM BPD criteria by systematically comparing LCA, FA, and different variants of FMM, in a large sample of help-seeking adolescents presenting with BPD features. The aim of the current study was twofold; first, to examine whether BPD in adolescence is a categorical, dimensional, or indeed mixed construct, and second, to characterize subgroups, if they existed, in terms of demographic, predisposing, and clinical variables at baseline and after one year of early intervention for BPD.

## Methods

### Participants

Data was collected from a consecutive sample recruited from a specialized outpatient service for adolescents presenting with risk-taking and self-harming behavior between April 2013 and November 2018. The service provides low-threshold initial contact, state-of-the-art diagnosis of BPD features, and evidence-based therapy for adolescents with emerging BPD. Inclusion criteria were age 12 to 17 years and any type of risk-taking or self-harming behavior (e.g., repeated non-suicidal self-injury (NSSI), suicide attempts, binge drinking, substance misuse, excessive gaming and internet use, risky sexual behavior, impulsive and delinquent behavior). Participants were only excluded for insufficient knowledge of the German language.

### Procedures

The study protocol was approved by the ethics committee of the Medical Faculty at the University of Heidelberg, Germany (S-449/2013). Written informed consent was obtained from participants who were ≥ 16 years of age. If participants were younger than 16 years of age, they were asked for written informed assent and their parents or legal guardians for written informed consent. Participants underwent a comprehensive assessment at baseline (T0) and at one-year follow-up (T1), including demographic information (e.g., age, sex), semi-structured clinical interviews, and questionnaires. The assessments were conducted by specially trained clinical psychologists. Participants were reimbursed for participating in the follow-up assessment (20 Euro).

### Measures

BPD symptoms and diagnosis were assessed using the *Structured Clinical Interview for DSM-IV Axis II Personality Disorders (SCID-II)* [30]. Note that the DSM-IV BPD criteria are the same as in the DSM-5 Section II. Each criterion is rated as 1 = *“not met”*, 2 = *“partly met”*, 3 = *“completely met”*. Additional variables used in the current study included conduct disorder (CD) and antisocial personality disorder (ASPD) diagnoses according to DSM-IV, assessed using the SCID-II; alcohol use disorder (AUD) and substance use disorder (SUD) according to DSM-IV and ICD-10, assessed using the structured Mini International Neuropsychiatric Interview for Children and Adolescents (MINI-KID) [31]; internet gaming disorder (IGD) according to DSM-5 [11], assessed using a structured clinical interview [32]; frequency of suicidal thoughts and attempts, and of NSSI over the past year, measured by the Self-Injurious Thoughts and Behaviors Interview (SITBI-G) [33]; severity of depression, assessed by the Children’s Depression Inventory (CDI) [34]; symptom burden, assessed by the Global Severity Index (GS) of the Symptom Check-List-90-R (SCL-90-R) [35]; illness severity, assessed by the Clinical Global Impression – Severity (CGI-S) scale [36]; clinical improvement, measured by the Clinical Global Impression – Improvement (CGI-I) scale [36]; psychosocial impairments, assessed by the DSM-IV Axis Five: Global Assessment of Functioning (GAF) [37]; quality of life, assessed by the KIDSCREEN-10 [38]; adverse childhood experiences, measured by the respective subscales for antipathy, neglect, physical abuse, and sexual abuse of the Childhood Experiences of Care and Abuse Questionnaire (CECA.Q) [39]; and personality traits, assessed by four higher-order personality dimensions – Emotional Dysregulation, Dissocial Behavior, Inhibition, and Compulsivity – of the Dimensional Assessment of Personality Pathology-Basic Questionnaire (DAPP-BQ) [40].

### Statistical analysis

First, we calculated the prevalence rates of the SCID-II BPD criteria as a marker of heterogeneity in the current sample. Second, we investigated the underlying latent structure by comparison of LCA, FA, and FMM. Third, we conducted post-hoc analyses to characterize the best fitting model using the additional measures. Step 1 and 3 were conducted using Stata/SE, version 16.0 [41]. Step 2 was performed using Latent GOLD® software, version 5.1 [42].

In order to investigate the latent structure of BPD (step 2), we applied LCA, FA, and FMM to the dummy-coded SCID-II BPD criteria. Ratings of 3 (*“completely met”*) were coded as 1 = *“present”*, ratings of 2 (*“partly met”*) and 1 (*“not met”*) were coded as 0 = *“absent”*. We closely followed the model building strategy proposed by Clark et al. [26]. First, we fitted LCA models with increasing numbers of classes. Based on the literature, we estimated LCA models with one to four classes [18–23]. Next, we modeled a single-factor confirmatory factor analysis (CFA) and the three-factor CFA reported by Sanislow et al. [24], which are the most replicated FA models in the BPD literature [10]. Finally, we fitted FMM with one factor and two or three classes, respectively. As shown below, this was the endpoint combination of number of classes and factors determined by our best fitting LCA and CFA models [26]. For each FMM, four variations with increasing measurement invariance were tested [26] (see SM Table 1 for further details on model specifications). Once the best fitting FMM was chosen, it was compared with the best fitting LCA and CFA models in order to determine the overall best fitting model [26].

The comparison of latent models was guided by statistical criteria, such as goodness-of-fit indices and entropy, and conceptual considerations [17]. To compare LCA models and FMM with different numbers of classes, the parametric bootstrapped likelihood ratio test (BLRT) [43] was used. Notably, when comparing FMM with the BLRT, only models that have the same parameterization, but differing numbers of classes can be compared. For comparison of FA models and among different model types (LCA, CFA, and FMM), the Bayesian Information Criterion (BIC) [44] and its sample size adjusted version (SABIC) [45] were used. The BIC is considered to be stricter than the SABIC [46, 47]. The BIC and SABIC are computed as a function of the log likelihood with a penalty for model complexity [17, 26, 48]. A difference of more than 10 in BIC values between two models indicates support for the model with the lower value [49]. In addition to the fit indices discussed, entropy was evaluated, which is a measure of the degree to which the latent classes are distinguishable and the precision with which individuals can be placed into classes. It ranges from 0 to 1, with higher values indicating clearer class separation. A value of ≥.80 is recommended, when participants shall be classified based on the “most likely class membership” resulting from LCA or FMM for further analysis [50].

Having identified the best fitting model, we examined the effects of sex and age as covariates [51], as these parameters may influence BPD symptom expression [52, 53]. In particular, we estimated the extent of the between-class and within-class variation of the best fitting model (see below) that was due to sex and age. This was done by regressing the class (corresponding with between-class variation) or the observed variables (corresponding with within-class variation) on sex and age [25] (further details on the covariate models are given in SM Fig. 1). Thereby, we fixed the age effect to be the same for all BPD criteria.

**Fig. 1:**
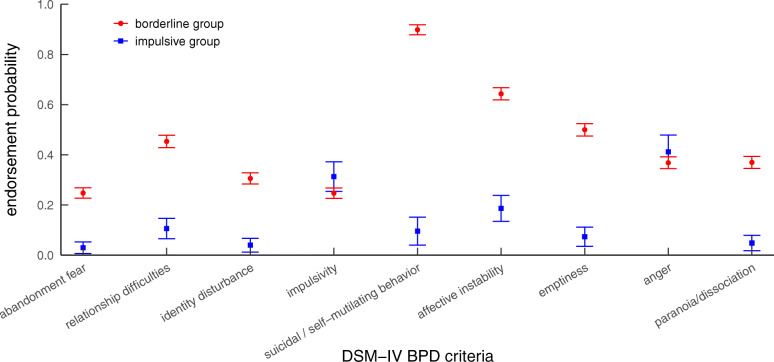
Latent class profile plot of the SCID-II BPD criteria. Dots/rectangles represent means, error bars represent standard errors. BPD = borderline personality disorder; DSM-IV = Diagnostic and Statistical Manual of Mental Disorders, fourth edition

Finally, post-hoc analyses (step 3) were conducted in order to characterize the classes identified by the best fitting model (see below). Therefore, participants were grouped according to their most likely latent class membership and compared with regard to demographic (age, sex), predisposing (adverse childhood experiences, personality traits), and clinical variables (BPD diagnosis and number of symptoms, CD/ASPD, AUD, SUD, IGD, NSSI, suicidal behavior, depression, symptom burden, quality of life, functional impairments, illness severity, and clinical improvement) at baseline and at follow-up. For the comparison of categorical variables, chi-square tests or Fisher’s exact tests, if expected cell counts were less than five, were used. For continuous variables, Mann-Withney *U* tests were used, when the assumption of normality was violated as indicated by a significant Shapiro-Wilk test. Effect sizes (Cramer’s *V* and Pearson’s correlation coefficient *r*) and corrected significance levels according to the method described by Benjamini and Hochberg [54] (in order to control for the increase of the type I error according to multiple testing) were reported for all group comparisons. Differences in continuous variables by group over time were tested using mixed-effects linear regression analyses. Measurement time point (T0, T1), latent class membership (borderline group vs. impulsive group), and their interaction were used as fixed effects, the study ID was used as a random effect. In case of missing values, the analyses were conducted on the subsample with complete data.

## Results

### Participants

Of *N* = 590 patients invited to take part in the study, *n* = 531 (90%) agreed to participate. Five (0.9%) who did not meet the age criteria, and 20 (3.8%) with missing information on the DSM-IV BPD criteria were excluded from the current study, resulting in a total sample of *N* = 506. The mean age of participants at baseline was 15.05 years (*SD* = 1.39), and the majority of the sample was female (*n* = 409; 80.8%). Table 1 gives an overview of primary diagnoses of the sample. Two-hundred and forty-six participants (48.6%) were assessed at one-year follow-up, 148 participants (29.3%) were lost to follow-up, and for 112 participants (22.1%) the follow-up assessment was still pending at the time of the analyses, due to the consecutive design of the study.

### Prevalence rates for the nine BPD criteria

Table 2 shows that there was variability in the endorsement of the nine DSM-IV BPD criteria. The self-injurious and suicidal behavior and affective instability criteria were met by the majority of the sample (58–78%). In contrast, the abandonment fear, identity disturbance, and impulsivity criteria were met by roughly a quarter of patients (22–26%). The endorsement of disturbed relationships, emptiness, anger, and paranoid ideation/dissociation criteria ranged between 32 and 44%.

**Table 2:** Prevalence rates of DSM-IV BPD criteria according to the SCID-II

Criterion		*N* (%)
1	Frantic efforts to avoid real or imagined abandonment	109 (21.5)
2	A pattern of unstable and intense interpersonal relationships	204 (40.3)
3	Identity disturbance	135 (26.7)
4	Impulsivity in at least two areas that are potentially self-damaging	130 (25.7)
5	Recurrent suicidal or self-mutilating behavior	395 (78.1)
6	Affective instability due to a marked reactivity of mood	292 (57.7)
7	Chronic feelings of emptiness	221 (43.7)
8	Inappropriate, intense anger or difficulty controlling ager	190 (37.5)
9	Transient, stress-related paranoid ideation or severe dissociative symptoms	163 (32.2)

*Notes. DSM-IV* Diagnostic and Statistical Manual of Mental Disorders, fourth edition;

*SCID-II* Structured Clinical Interview for DSM-IV Axis II Personality Disorders (SCID-II)

### Comparison of LCA, CFA, and FMM

Table 3 presents the fit indices for the LCA, CFA, and FMM models. First, we compared LCA models with one to four classes. The two- and three-class solutions showed the best model fit according to the BIC, while the SABIC decreased as the number of classes increased. With only a small difference in the BIC values between the two- and three-class solutions (< 10), the three-class solution was supported by the significant BLRT, but had a lower entropy value (0.66 vs. 0.74, respectively). Second, we estimated a single-factor CFA model and the three-factor CFA according to Sanislow et al. [24]. Based on BIC and SABIC, the CFA model with one factor outperformed the CFA model with three factors. In line with the model building strategy proposed by Clark et al. [26], we used the combination of one factor and three classes as the ending point for the FMM fitting procedure. Accordingly, we estimated two FMM with one factor and two or three classes, respectively, and tested four variants of each with increasing measurement invariance (see SM Table 1). Considering BIC and SABIC, variant three of the two-classes/one-factor FMM was superior to the competing models, but its entropy value was low (0.54). The non-significant BLRT supported the selection of the two-classes/one-factor FMM-3 over the three-classes/one-factor FMM-3. Finally, we compared the fit indices across model types. According to BIC and SABIC, the two-class/one-factor FMM-3 fitted the DSM-IV BPD criteria best. However, its entropy value was substantially lower than the recommended threshold value of 0.80, indicating that class assignment based on the model is problematic.

**Table 3:** Model fit comparisons for latent class analysis (LCA), confirmatory factor analysis (CFA), and factor mixture models (FMM).

Model	LL	No. of par.	BIC	SABIC	Entropy	BLRT
LCA						
One-class	− 2797.12	9	5650.28	5621.71	1	
Two-class	− 2542.70	19	5203.71	5143.40	0.74	≤.001***
Three-class	− 2508.00	29	5196.57	5104.52	0.66	≤.001***
Four-class	− 2488.52	39	5219.87	5096.09	0.63	≤.001***
CFA						
One-factor	− 2518.92	18	5149.91	5092.77	N/A	N/A
Three-factor^1^	− 2515.43	21	5161.62	5094.97	N/A	N/A
FMM						
Two-class, one-factor						
FMM-1	−2542.70	19	5203.71	5143.40	0.74	
FMM-2	− 2517.43	21	5165.61	5098.96	0.08	
FMM-3	− 2481.07	29	5142.70	5050.65	0.54	
FMM-4	− 2472.06	37	5174.51	5057.07	0.55	
Three-class, one-factor						
FMM-1	− 2521.74	21	5174.23	5107.58	0.66	≤.001***
FMM-2	−2515.70	24	5180.83	5104.65	0.28	.08
FMM-3	−2472.28	40	5193.63	5066.66	0.53	.24
FMM-4	− 2450.36	56	5249.41	5071.66	0.60	.06

*Notes. BIC* Bayesian Information Criterion, *BLRT* parametric bootstrap likelihood ratio test, *LL*

Log-Likelihood, *FFM* factor mixture model, *SABIC* sample size adjusted BIC

^1^ according to Sanislow et al. [24]

Table 4 shows the fit indices for the tested covariate models that explored the effects of sex and/or age on the best-fitting two-class/one-factor FMM-3 (see also SM Fig. 1 for a graphical illustration of the covariate models). We started by testing the effect of sex on the latent class variable and the DSM-IV BPD criteria, respectively. Compared with the model without any covariate, the model that included an effect of sex on the latent class variable demonstrated lower BIC and SABIC values, indicating better model fit, and improved entropy (0.76). In contrast, the model that regressed the DSM-IV BPD criteria on sex performed worse in terms of BIC and entropy, compared with the model without any covariate. Next, we tested the additional effect of age on the latent class variable and the DSM-IV BPD criteria, respectively. The model that included a direct effect of sex on the latent class membership and an additional effect of age on the DSM-IV BPD criteria outperformed all other models in terms of both fit indices and entropy. With an entropy value of 0.79, the recommended threshold of 0.80 for post-hoc analyses based on latent class membership was nearly reached. In this final model, the effect of sex on the latent class variable was significant, with r = − 1.00, Wald *χ*^*2*^(1) = 39.32, *p* = ≤.001. Additionally, age significantly affected the DSM-IV BPD criteria, with r = 0.19, Wald *χ*^*2*^(1) = 60.59, *p* = ≤.001.

**Table 4:** Model fit comparisons for two-class/one-factor FMM-3 with different covariate effects

Model		LL	No. of parameters	BIC	SABIC	Entropy
1	No covariates^1^	−2481.07	29	5142.70	5050.65	0.54
2	Model 1 + sex on latent class	− 2434.62	30	5056.04	4960.82	0.76
3	Model 1 + sex on observed variables	− 2416.15	38	5068.90	4948.29	0.44
4	Model 2 + age on latent class	−2434.22	31	5061.47	4963.07	0.74
5	Model 2 + age on observed variables	− 2403.71	31	5000.44	4902.05	0.79

*Notes. BIC* Bayesian Information Criterion, *BLRT* parametric bootstrap likelihood ratio test; LL =

Log-Likelihood, *FFM* factor mixture model, *SABIC* sample size adjusted BIC

^1^ corresponds with the two-class/one-factor FMM-3 from Table 1

### Characterizing the best fitting model

Figure 1 presents the latent class profiles based on the DSM-IV BPD criteria. Class 1 was the larger class (85%) and characterized by relatively high probabilities for all BPD criteria, ranging from 0.25 for criterion 4 (impulsivity) to 0.90 for criterion 5 (self-injurious and suicidal behavior). Class 2 was the smaller class (15%) and characterized by relatively low probabilities for all BPD criteria (≤ 0.19), except from criteria 4 (impulsivity; 0.31) and 8 (anger; 0.41). The two classes significantly differed in the likelihood of occurrence of all symptoms, except from impulsivity (*p* = .52) and anger (*p* = .80). In accordance with the two subtypes of the emotionally unstable personality disorder according to the ICD-10 [55], the two classes were labeled as “borderline group” and “impulsive group”.

Based on the most likely latent class membership of the best fitting model, 439 (86.8%) adolescents belonged to the borderline group, and 67 (13.2%) to the impulsive group. Full results of the group-wise comparisons for demographic, predisposing, and clinical variables at baseline and one-year follow-up are given in Table 5. The borderline group included more females and younger patients compared with the impulsive group who consisted of more males and older patients. In terms of predisposing factors, the borderline group reported more frequently sexual abuse, antipathy, or neglect during early childhood, and scored higher on Emotional Dysregulation and Inhibitedness personality traits, while the impulsive group scored higher on the Dissocial Behavior personality trait. Regarding clinical characteristics at baseline, the borderline group was more frequently diagnosed with full-threshold BPD, met a greater number of BPD criteria, reported more frequent suicidal thoughts, suicidal attempts, and NSSI in the past year, showed more depressive symptoms, reported a higher symptom burden and lower quality of life, showed greater functional impairments, and was rated as overall more severely ill, compared with the impulsive group. In contrast, the impulsive group was more often diagnosed with CD or ASPD, SUD, and IGD, compared with the borderline group. Effect sizes were small to moderate. At one-year follow-up, clinical differences between groups remained stable, except that the group differences in SUD, IGD, functional impairments and overall illness severity disappeared. Mixed-effects linear regression analyses (see Table 6) demonstrated a significant reduction of number of BPD symptoms, suicidal thoughts, NSSI, depression, and symptom burden, and a significant increase of quality of life in the borderline group, as well as a significant decrease of functional impairments and overall illness severity in both groups over time. The measurement time point x latent class membership interaction was not significant for any of the outcome variables.

**Table 5:** Group differences in demographic, predisposing, and clinical variables at baseline (T0) and follow-up (T1)

	Borderline type (*n* = 439)			Impulsive type (*n* = 67)			Group comparison			
	*n* (%)	*M* (SD)	Mdn	*n* (%)	*M* (SD)	Mdn	Statistics	*p*	BH	Effect size
DEMOGRAPHIC VARIABLES										
Male (*n* = 506)	34 (7.7)			63 (94.0)			χ^2^(1) = 279.30	<.001***	s.	*V* = .74
Age (*n* = 506)		14.95 (1.36)	15.00		15.72 (1.40)	16.00	*U =* 9866.50	<.001***	s.	*r* = .19
PREDISPOSING VARIABLES										
CECA.Q antipathy (*n* = 457)	227 (57.3)			18 (29.5)			χ^2^(1) = 16.44	<.001***	s.	*V* = .19
CECA.Q neglect (*n* = 457)	175 (44.2)			17 (27.9)			χ^2^(1) = 5.78	.016*	s.	*V* = .11
CECA.Q physical abuse (*n* = 453)	106 (27.0)			14 (23.0)			χ^2^(1) = .453	.501	n.s.	*V* = .03
CECA.Q sexual abuse (*n* = 457)	94 (23.7)			5 (8.2)			χ^2^(1) = 7.52	.006**	s.	*V* = .13
DAPP-BQ Emotional Dysregulation (*n* = 453)		383.56 (62.25)	388.00		304.53 (69.23)	318.00	*U* = 18,228.50	<.001***	s.	*r* = .32
DAPP-BQ Dissocial Behavior (*n* = 452)		201.64 (42.67)	200.00		219.81 (43.65)	235.00	*U* = 8316.00	.001**	s.	*r* = .15
DAPP-BQ Inhibitedness (*n* = 437)		109.41 (27.01)	105.00		93.64 (21.45)	89.00	*U* = 14,635.50	<.001***	s.	*r* = .22
DAPP-BQ Compulsivity (*n* = 460)		42.43 (10.86)	42.00		43.28 (10.50)	43.00	*U* = 11,054.00	.646	n.s.	*r* = .02
CLINICAL VARIABLES										
SCID-II BPD										
T0 (n = 506)	183 (41.7)			1 (1.5)				<.001***	s.	*V* = .28
T1 (*n* = 235)	72 (34.1)			0 (0)				<.001***	s.	*V* = .22
No. SCID-II BPD criteria										
T0 (n = 506)		3.97 (2.24)	4.00		1.40 (1.38)	1.00	*U* = 24,285.00	<.001***	s.	*r* = .34
T1 (n = 235)		3.44 (2.22)	3.00		1.50 (1.18)	2.00	*U* = 3808.50	<.001***	s.	*r* = .25
SCID-II CD										
T0 (*n* = 502)	59 (13.6)			36 (53.7)			χ^2^(1) = 61.05	<.001***	s.	*V* = .35
T1 (*n* = 236)	22 (10.4)			7 (29.2)			χ^2^(1) = 7.06	.008**	s.	*V* = .17
SCID-II ASPD										
T0 (*n* = 496)	21 (4.9)			12 (18.8)			χ^2^(1) = 17.31	<.001***	s.	*V* = .19
T1 (*n* = 234)	3 (1.4)			4 (16.7)				002**	s.	*V* = .27
MINI-KID AUD										
T0 (*n* = 148)	36 (29.5)			12 (46.2)			χ^2^(1) = 2.71	.100	n.s.	*V* = .1 4
T1 (*n* = 75)	28 (45.9)			5 (35.7)			χ^2^(1) = 0.48	.489	n.s.	*V* = .08
MINI-KID SUD										
T0 (*n* = 126)	26 (26.3)			17 (63.0)			χ^2^(1) = 12.71	<.001***	s.	*V* = .32
T1 (*n* = 42)	23 (69.7)			7 (77.8)			χ^2^(1) = 0.23	.634	n.s.	*V* = .07
IGD										
T0 (*n* = 250)	1 (0.5)			4 (9.8)				.003**	s.	*V* = .25
T1 (*n* = 137)	1 (0.8)			0 (0)				1.000	n.s.	*V* = .03
SITBI no. suicidal thoughts past year										
T0 (*n* = 501)		100.39 (105.58)	70.00		16.03 (49.21)	0.00	*U* = 24,698.50	<.001***	s.	*r* = .36
T1 (*n* = 237)		86.90 (90.48)	54.00		20.83 (37.14)	1.00	*U* = 3954.00	<.001***	s.	*r* = .27
SITBI no. suicidal attempts past year										
T0 (*n* = 504)		1.96 (9.28)	0.00		0.13 (0.46)	0.00	*U* = 19,503.50	<.001***	s.	*r* = .22
T1 (*n* = 237)		0.61 (1.61)	0.00		0.08 (0.28)	0.00	*U* = 3072.00	.034*	s.	*r* = .14
SITBI no. NSSI past year										
T0 (*n* = 500)		71.44 (82.77)	45.00		10.14 (26.80)	0.00	*U* = 23,978.50	<.001***	s.	*r* = .36
T1 (*n* = 236)		50.39 (64.17)	24.50		7.63 (20.99)	0.00	*U* = 4128.50	<.001***	s.	*r* = .30
CDI										
T0 (*n* = 458)		30.25 (9.30)	31.00		17.13 (8.40)	16.00	*U* = 20,508.00	<.001***	s.	*r* = .35
T1 (*n* = 208)		23.24 (11.77)	23.50		15.19 (10.72)	10.50	*U = 2134.00*	.010**	s.	*r = .17*
SCL-90-R GSI										
T0 (*n* = 467)		1.68 (0.71)	1.74		0.80 (0.69)	0.70	*U* = 20,187.50	<.001***	s.	*r* = .33
T1 (*n* = 236)		1.26 (0.81)	1.15		0.64 (0.64)	0.38	*U* = 3459.00	<.001***	s.	*r* = .22
KIDSSCREEN-10										
T0 (*n* = 417)		33.99 (6.62)	33.79		44.81 (10.06)	43.35	*U* = 3109.50	<.001***	s.	*r* = .33
T1 (*n* = 186)		38.93 (9.26)	37.42		49.22 (11.52)	48.29	*U* = 524.50	.001**	s.	*r* = .22
GAF										
T0 (*n* = 422)		47.46 (11.45)	48.00		55.28 (14.04)	51.00	*U* = 7130.00	<.001***	s.	*r* = .21
T1 (n = 236)		61.91 (13.59)	60.00		65.96 (14.69)	60.00	*U* = 2166.00	.231	n.s.	*r* = .08
CGI-S										
T0 (*n* = 480)		5.10 (0.91)	5.00		4.56 (1.11)	5.00	*U* = 16,959.00	<.001***	s.	*r* = .17
T1 (*n* = 235)		3.65 (1.33)	4.00		3.13 (1.36)	3.50	*U* = 3056.50	.087	n.s.	*r* = .11
CGI-I (*n* = 232)		2.74 (1.13)	2.00		2.54 (1.22)	2.00	*U* = 2742.50	.398	n.s.	*r* = .06

*Notes. AUD* alcohol use disorder, *ASPD* antisocial personality disorder, *BH* Benjamini-Hochberg correction, *BPD* borderline personality disorder, *CD* Conduct Disorder, *CDI* Children’s Depression Inventory, *CECA. Q* Childhood Experience of Care and Abuse Questionnaire, *CGI-S/−I* Clinical Global Impression Severity / Improvement scale, *DAPP-BQ* Dimensional Assessment of Personality Pathology Basic Questionnaire, *GAF* DSM-IV Axis Five Global Assessment of Functioning, *IGD* internet gaming disorder, *KIDSCREEN-10* health-related quality of life, *MINI-KID* Mini International Neuropsychiatric Interview for Children and Adolescents, *NSSI* non-suicidal self-injury, *SCID-II* Structured Clinical Interview for DSM-IV Axis II Personality Disorders, *SITBI* Self-Injurious Thoughts and Behaviors Interview; *SCL-90-R GSI* Symptom-Checklist-90-Revised Global Severity Index, *SUD* substance use disorder

**Table 6:** Group differences in clinical improvement over time

Variable	predictor	Contr. (Std. Err)	*p*	95% CI
No. SCID-II BPD criteria	Borderline type: T0 vs T1	−0.55 (0.14)	<.001***	[−0.87, − 0.24]
	Impulsive type: T0 vs T1	−0.10 (0.41)	.960	[−1.02, 0.82]
	Interaction term	−0.45 (0.43)	.303	[−0.40, 1.30]
SITBI no. suicidal thoughts past year	Borderline type: T0 vs T1	−16.32 (6.76)	.031*	[−31.44, − 1.20]
	Impulsive type: T0 vs T1	−3.20 (19.63)	.983	[− 47.11, 40.71]
	Interaction term	13.12 (20.77)	.528	[−27.58, 53.82]
STIBI no. suicidal attempts past year	Borderline type: T0 vs T1	−0.58 (0.26)	.050	[−1.17, 0.00]
	Impulsive type: T0 vs T1	−0.01 (0.78)	1.000	[−1.74, 1.73]
	Interaction term	0.58 (0.82)	.479	[−1.02, 2.18]
SITBI no. NSSI past year	Borderline type: T0 vs T1	−23.61 (5.23)	<.001***	[−35.31, −11.91]
	Impulsive type: T0 vs T1	−2.30 (15.28)	.986	−36.47, 31.87]
	Interaction term	21.31 (16.15)	.187	[−10.34, 52.96]
CDI	Borderline type: T0 vs T1	−6.93 (0.72)	<.001***	[−8.54, −5.32]
	Impulsive type: T0 vs T1	−2.46 (2.39)	.513	[−7.80, 2.88]
	Interaction term	4.47 (2.49)	.073	[−0.42, 9.36]
SCL-90-R GSI	Borderline type: T0 vs T1	−0.43 (0.05)	<.001***	[−0.54, − 0.32]
	Impulsive type: T0 vs T1	−0.21 (0.15)	.296	[−0.55, − 0.13]
	Interaction term	0.22 (0.16)	.161	[−0.09, 0.53]
KIDSCREEN-10	Borderline type: T0 vs T1	4.86 (0.64)	<.001***	[3.43, 6.30]
	Impulsive type: T0 vs T1	3.48 (2.22)	.222	[−1.49, 8.46]
	Interaction term	− 1.38 (2.32)	.551	[−5.92, 3.16]
GAF	Borderline type: T0 vs T1	14.61 (0.94)	<.001***	[12.51, 16.71]
	Impulsive type: T0 vs T1	10.59 (2.63)	<.001***	[4.70, 16.48]
	Interaction term	−4.02 (2.80)	.151	[−9.51, 1.47]
CGI-S	Borderline type: T0 vs T1	−1.45 (0.08)	<.001***	[−1.63, − 1.27]
	Impulsive type: T0 vs T1	−1.38 (0.23)	<.001***	[−1.90, −0.87]
	Interaction term	0.06 (0.24)	0.793	[−0.41, 0.54]

*Notes. CGI-S* Clinical Global Impression Severity scale, *GAF* DSM-IV Axis Five Global Assessment of Functioning, *KIDSCREEN-10* health-related quality of life, *NSSI* non-suicidal self-injury, *SCID-II* Structured Clinical Interview for DSM-IV Axis II Personality Disorders, *SITBI* Self-Injurious Thoughts and Behaviors Interview, *SCL-90-R GSI* Symptom-Checklist-90-Revised Global Severity Index

## Discussion

In face of the clinically challenging heterogeneous presentation of BPD and the enduring scientific debate about whether the heterogeneity can be best explained by categorical or dimensional differences between individuals, the current study applied LCA (investigating qualitatively distinct subtypes), FA (investigating dimensional differences) and FMM (allowing a latent structure to have both categorical and dimensional aspects) to the DSM-IV BPD criteria in a sample of adolescent outpatients with risk-taking and/or self-harming behavior. The main result that emerged from the study was that help-seeking adolescents with BPD features are best represented as two qualitatively distinct subgroups, with sex significantly explaining group membership, and both a latent factor and age explaining heterogeneity within groups. As the latent factor in our best fitting model explained within-class variability only, the two identified groups cannot be compared with regard to mean differences in the factor. As implied by the class-varying item thresholds, the two groups were based on the responses to the BPD criteria rather than the factor mean and variance [26]. There was a majority group with relatively high probabilities for all BPD criteria (“the borderline group”), and a minority group with relatively low probabilities for all BPD criteria, except from impulsivity and anger (“the impulsive group”). The class-varying covariance matrix allowed for different levels of heterogeneity within each class, resulting in the borderline group having a greater range of symptoms compared with the impulsive group. Considering sex and age as covariates significantly improved the model fit, indicating that these variables should be taken into account when explaining heterogeneity of BPD among adolescents. Being female was associated with a greater likelihood of belonging to the borderline group, being male with a greater likelihood of belonging to the impulsive group. Within each group, older adolescents were more likely to meet a BPD criterion than younger adolescents, which is in line with the epidemiological finding that BPD first emerges during adolescence and peaks during early adulthood [2]. The two identified groups demonstrated meaningful differences in predisposing factors and clinical variables, supporting their validity.

From a developmental perspective [56], it could be argued that the borderline group included individuals who had experienced emotional abuse / neglect or sexual abuse early in life and then developed a personality characterized by high negative emotionality, stress sensitivity, and social inhibition [40, 57], which in turn made them more susceptible to severe psychopathology, functional impairments, and life dissatisfaction. In contrast, the impulsive group may have consisted of people characterized by an attitude of lack of regard for others [40, 57], which in turn predisposed them to dissocial behavior (as captured by the CD diagnosis) and substance-related and behavioral addictions, resulting in a phenotype resembling ASPD in adulthood. The developmental pathway appeared to be crucially influenced by sex, with females rather belonging to the borderline group and males to the impulsive group. The question arises whether the impulsive group actually represents a “true” (sub-threshold) BPD group or whether it would be better described as a group of adolescents with predominantly CD who are at high risk of developing ASPD in adulthood. This interpretation is supported by the fact that CD in adolescence is an established precursor of ASPD in adulthood [58]. Further, evidence indicates that BPD and ASPD share common biological vulnerabilities (e.g., trait impulsivity derived from dopaminergic and serotonergic dysfunctions) and environmental risk factors (e.g., disrupted attachment, abuse and neglect), with sex moderating the phenotypical expression of biology x environment interactions to produce BPD overproportionately in females and ASPD overproportionately in males [59]. For instance, some high risk genes may confer differential vulnerability to internalizing behaviors among girls versus externalizing behavior among boys. Additionally, deviant peer group affiliations may emerge during adolescence, leading girls to become exposed to self-injurious behaviors of peers and boys to delinquent behaviors [59]. Future examination of the stability of the two identified adolescent groups over time is needed.

Our findings are most consistent with the LCA results reported by Fossati et al. [21] and Thatcher et al. [23]. Both reported an impulsive class that endorsed symptoms of impulsivity and anger only, along with two [21] or three [23] BPD classes differing in severity. Comparably to our findings, the impulsive group in Thatcher et al. [23]‘s study included an overproportionally large number of males and was characterized by high rates of CD, while the severe BPD group was distinguished by high rates of depression. Our findings stand in contrast to previous studies suggesting that the heterogeneous clinical presentation of BPD can be best understood in terms of individual differences on a single underlying trait (“BPD-ness”) or subgroups that lie on a continuum of BPD severity [19, 20, 22, 28, 29].

Several methodological reasons may account for these diverging results. First, the majority of studies did either apply LCA or FA on the diagnostic criteria when investigating the latent structure of BPD [18, 19, 22, 23], while we systematically compared LCA, FA, and FMM. Second, only a few studies have systematically explored the effects of covariates, such as sex and age. There have been mixed results, with two studies reporting that females were more likely than males to belong to the class with more BPD criteria [19, 20], and one study reporting no sex difference [28]. To the best of our knowledge, the impact of age has only been examined in one study [20] that found that the probability of belonging to the borderline group declined with increasing age until the age of 27, from which the probability increased. Our results confirm that sex might have an important impact on latent class membership, with females having a greater likelihood of belonging to the borderline group than males. We could not replicate a direct effect of age on latent group membership, but found that age explains within-class variability, with the probability of endorsing a BPD criterion being higher with increasing age. Third, the studies included various clinical and community samples, with well-known differences in prevalence rates for females and males. In community samples, the sex ratio is 1:1, while clinical samples usually show three times more females than males with the disorder [1]. Forth and probably most importantly, because BPD presents differently across the lifespan [15], the majority of studies have examined adults with mean ages ranging between 20 and 42 years [19–23, 28, 29], while our sample consisted of adolescents with a mean age of 15 years. We are aware of three studies investigating subtypes of BPD in adolescence. Two of them identified two subgroups (based on either the personality pattern scales from the Millon Adolescent Clinical Inventory [60], or the Shedler-Westen Assessment Procedure-200 for Adolescents [61]) that were clearly gendered and differed regarding the internalizing-externalizing dimensions of psychopathology [62, 63], with internalizing psychopathology being more common among females and externalizing psychopathology being more common among males [64, 65]. The third study examined females only and identified four groups (based on the Borderline Personality Questionnaire [66]) with an increasing number of BPD symptoms and distinct patterns of comorbidities [18]. Our results are consistent with the finding of a more female, internalizing group, and a more male, externalizing group.

Clinically, our findings have several important implications. First, they are in favor of early assessment and treatment of borderline features among help-seeking adolescents, even if they are below the diagnostic threshold [2, 7, 9], as they are associated with co-occurring psychopathology, functional impairments, and high emotional burden [67]. The borderline group based on latent group membership was more inclusive than the DSM-IV, with 42% meeting the diagnostic threshold of five DSM-IV criteria at baseline, and the average number of BPD criteria being nearly four (see Table 5). This finding is in line with an adult study reporting that the borderline latent class was more inclusive than diagnoses based on the DSM-III-R threshold (which is the same as in DSM-IV and − 5) [20]. Thus, our results add to the evidence suggesting that the DSM BPD threshold is too restrictive to adequately conceptualize the borderline construct in adolescents [68] and adults [20]. Second, the low rate of males in our sample along with the well-known 1:1 sex ratio for BPD in adult community samples [1] implies that many young males with BPD features such as impulsivity and anger may not access mental health services, but turn up on other services’ doorsteps, including police services and courts. An integrated treatment approach that involves collaboration between services is needed to improve treatment access and engagement for this particular group. Third, mixed-effects linear regression analyses did not find a group difference in clinical improvement over time, indicating that both groups benefited from the received treatment that included elements from cognitive behavioral therapy and dialectical behavioral therapy [69, 70]. However, due to the short follow-up period and the substantial amount of missing data in the current study, this finding has to be considered as preliminary. Future studies examining between and within group variability in clinical changes of the two identified groups over a longer period of time are required to clarify whether or not group-specific treatment adaptations could be beneficial.

The strengths of the current study include a large representative sample of help-seeking adolescents with BPD features, the structured assessment of BPD pathology by trained psychologists, the systematic comparison of different latent models according to the procedure proposed by Clark et al. [26], the consideration of sex and age as covariates in the latent models, and the validation of the identified latent structure using external variables. The study has several limitations that ought to be considered. First, the sample was drawn from adolescents seeking help from an outpatient service for risk-taking or self-harming behavior. Consequently, “acute” symptoms as assessed by DSM-IV BPD criterion 4 (impulsive behaviors such as binge drinking, substance misuse or risky sexual behavior) and 5 (recurrent suicidal or self-mutilating behavior) may be overrepresented in the sample and have contributed to the identification of the “impulsive group” in the current study. Second, there was a substantial amount of missing values in the variables used for post-hoc comparisons of the latent classes. Reasons for the missing values include the nature of the consecutive sample, the omission of questions, and the introduction of additional measures during the running study. Third, as Latent GOLD® does not provide common fit indices for comparison of FA models (e.g., Comparative Fit Index or Root Mean Square Error of Approximation), our selection of the best fitting CFA was based on the BIC and SABIC only. Last, as BPD criteria wax and wane over time, it has been argued that subtyping individuals with BPD features according to underlying pathological mechanisms may be a more promising approach [29, 65, 71].

## Conclusions

The current study provides evidence that the heterogeneous symptomatology of help-seeking adolescents with BPD features can be best understood in terms of two qualitatively distinct subgroups: One group that primarily includes females, is associated with internalizing psychopathology, and resembles the borderline type of the ICD-10 emotionally unstable personality disorder; and one group that primarily consists of males, is associated with externalizing psychopathology, and resembles the impulsive type of the ICD-10 emotionally unstable personality disorder. More research is needed to examine the diagnostic stability of the impulsive group in the long-term, and potential differential treatment indications for the two groups.

## Supplementary Information

**Additional file 1: Table S1.** Specification and graphic illustration of FMM variants according to Clark et al. (2013). **Figure S1.** Graphical illustration of the best fitting two-class/one-factor FMM-3 model with different covariate effects

## Data Availability

The datasets used and/or analyzed during the current study are available from the corresponding author on reasonable request.

## Abbreviations

ASPD: Antisocial Personality Disorder
AUD: Alcohol Use Disorder
BIC: Bayesian Information Criterion
BLRT: Bootstrapped Likelihood Ratio Test
BPD: Borderline Personality Disorder
CDI: Children’s Depression Inventory
CECA.Q: Childhood Experiences of Care and Abuse Questionnaire
CFA: Confirmatory Factor Analysis
CGI-I: Clinical Global Impression – Improvement
CGI-S: Clinical Global Impression – Severity
DAPP-BQ: Dimensional Assessment of Personality Pathology-Basic Questionnaire
DSM: Diagnostic and Statistical Manual of Mental Disorders
FA: Factor Analysis
FFM: Factor Mixture Models
GAF: Global Assessment of Functioning
ICD: International Statistical Classification of Diseases and Related Health Problems
IGD: Internet Gaming Disorder
LCA: Latent Class Analysis
MINI-KID: Mini International Neuropsychiatric Interview for Children and Adolescents
NSSI: Non-Suicidal Self-Injury
SABIC: Sample size Adjusted Bayesian Information Criterion
SCID-II: Structured Clinical Interview for DSM-IV Axis II Personality Disorders (SCID-II)
SCL-90-R: Symptom Check-List-90-R
SITBI-G: Self-Injurious Thoughts and Behaviors Interview German
SUB: Substance Use Disorder
SM: Supplementary Material

## References

[1] Gunderson JG, Herpertz SC, Skodol AE, Torgersen S, Zanarini MC (2018). Borderline personality disorder. Nat Rev Dis Primer.

[2] Kaess M, Brunner R, Chanen AM (2014). Borderline personality disorder in adolescence. Pediatrics..

[3] Gunderson JG (2011). Ten-year course of borderline personality disorder: psychopathology and function from the collaborative longitudinal personality disorders study. Arch Gen Psychiatry.

[4] Videler AC, Hutsebaut J, Schulkens JEM, Sobczak S, van Alphen SPJ (2019). A life span perspective on borderline personality disorder. Curr Psychiatry Rep..

[5] Winsper C, Marwaha S, Lereya ST, Thompson A, Eyden J, Singh SP (2015). Clinical and psychosocial outcomes of borderline personality disorder in childhood and adolescence: a systematic review. Psychol Med.

[6] Chanen AM, Sharp C, Hoffman P (2017). Global Alliance for prevention and early intervention for borderline personality disorder. Prevention and early intervention for borderline personality disorder: a novel public health priority. World Psychiatry.

[7] Sharp C, Fonagy P (2015). Practitioner review: borderline personality disorder in adolescence - recent conceptualization, intervention, and implications for clinical practice. J Child Psychol Psychiatry.

[8] Chanen AM (2015). Borderline personality disorder in young people: are we there yet?: borderline personality disorder. J Clin Psychol.

[9] Fonagy P, Speranza M, Luyten P, Kaess M, Hessels C, Bohus M (2015). ESCAP expert article: borderline personality disorder in adolescence: an expert research review with implications for clinical practice. Eur Child Adolesc Psychiatry.

[10] Michonski JD. The Underlying Factor Structure of DSM criteria in Youth BPD. In: Sharp C, Tackett JL, editors. Handbook of Borderline Personality Disorder in Children and Adolescents. New York: Springer New York; 2014 [cited 2019 Jan 20]. p. 35–48. Available from: http://link.springer.com/10.1007/978-1-4939-0591-1_4

[11] American Psychiatric Association (2013). Diagnostic and statistical manual of mental disorders.

[12] Tyrer P, Mulder R, Kim Y-R, Crawford MJ (2019). The development of the ICD-11 classification of personality disorders: an amalgam of science, pragmatism, and politics. Annu Rev Clin Psychol.

[13] Benson KT, Donnellan MB, Morey LC (2017). Gender-related differential item functioning in DSM-IV/DSM-5-III (alternative model) diagnostic criteria for borderline personality disorder. Personal Disord Theory Res Treat.

[14] Aggen SH, Neale MC, Røysamb E, Reichborn-Kjennerud T, Kendler KS (2009). A psychometric evaluation of the DSM-IV borderline personality disorder criteria: age and sex moderation of criterion functioning. Psychol Med.

[15] Hutsebaut J, Videler AC, Verheul R, Van Alphen SPJ (2019). Managing borderline personality disorder from a life course perspective: clinical staging and health management. Personal Disord Theory Res Treat.

[16] Hallquist MN, Wright AGC (2014). Mixture modeling methods for the assessment of Normal and abnormal personality, part I: cross-sectional models. J Pers Assess.

[17] Masyn KE, Henderson CE, Greenbaum PE (2010). Exploring the latent structures of psychological constructs in social development using the dimensional-categorical Spectrum: the dimensional-categorical Spectrum. Soc Dev.

[18] Slavin-Stewart C, Boylan K, Burke JD (2018). Subgroups of adolescent girls with borderline personality disorder symptoms. J Personal Disord.

[19] Bornovalova MA, Levy R, Gratz KL, Lejuez CW (2010). Understanding the heterogeneity of BPD symptoms through latent class analysis: initial results and clinical correlates among inner-city substance users. Psychol Assess.

[20] Clifton A, Pilkonis PA (2007). Evidence for a single latent class of diagnostic and statistical manual of mental disorders borderline personality pathology. Compr Psychiatry.

[21] Fossati A, Maffei C, Bagnato M, Donati D, Namia C, Novella L (1999). Latent structure analysis of DSM-IV borderline personality disorder criteria. Compr Psychiatry.

[22] Shevlin M, Dorahy M, Adamson G, Murphy J (2007). Subtypes of borderline personality disorder, associated clinical disorders and stressful life-events: a latent class analysis based on the British psychiatric morbidity survey. Br J Clin Psychol.

[23] Thatcher DL, Cornelius JR, Clark DB (2005). Adolescent alcohol use disorders predict adult borderline personality. Addict Behav.

[24] Sanislow CA, Grilo CM, Morey LC, Bender DS, Skodol AE, Gunderson JG (2002). Confirmatory factor analysis of DSM-IV criteria for borderline personality disorder: findings from the collaborative longitudinal personality disorders study. Am J Psychiatry.

[25] Lubke GH, Muthén B (2005). Investigating population heterogeneity with factor mixture models. Psychol Methods.

[26] Clark SL, Muthén B, Kaprio J, D’Onofrio BM, Viken R, Rose RJ. Models and strategies for factor mixture analysis: an example concerning the structure underlying psychological disorders. Struct Equ Model Multidiscip J 2013 1;20(4). 10.1080/10705511.2013.824786 PMC3844130 24302849

[27] Putnick DL, Bornstein MH (2016). Measurement invariance conventions and reporting: the state of the art and future directions for psychological research. Dev Rev.

[28] Conway C, Hammen C, Brennan P (2012). A comparison of latent class, latent trait, and factor mixture models of *DSM-IV* borderline personality disorder criteria in a community setting: implications for *DSM-5*. J Personal Disord.

[29] Hallquist MN, Pilkonis PA (2012). Refining the phenotype of borderline personality disorder: diagnostic criteria and beyond. Personal Disord Theory Res Treat.

[30] First M, Spitzer R, Gibbon M, Williams J, Benjamin L (1994). Structured clinical interview for DSM-IV Axis II personality disorders (SCID-II).

[31] Sheehan DV, Sheehan KH, Shytle RD, Janavs J, Bannon Y, Rogers JE (2010). Reliability and validity of the Mini international neuropsychiatric interview for children and adolescents (MINI-KID). J Clin Psychiatry.

[32] Kaess M, Whittle S, O’Brien-Simpson L, Allen NB, Simmons JG (2018). Childhood maltreatment, pituitary volume and adolescent hypothalamic-pituitary-adrenal axis – evidence for a maltreatment-related attenuation. Psychoneuroendocrinology..

[33] Fischer G, Ameis N, Parzer P, Plener PL, Groschwitz R, Vonderlin E (2014). The German version of the self-injurious thoughts and behaviors interview (SITBI-G): a tool to assess non-suicidal self-injury and suicidal behavior disorder. BMC Psychiatry.

[34] Stiensmeier-Pelster J, Schürmann M, Duda K. Depressionsinventar für Kinder und Jugendliche (DIKJ) (2 ed). Göttingen: Hogrefe; 2000.

[35] Franke GH (2002). SCL-90-R. Die Symptom-Checkliste von L.R. Derogatis (2. Auflage).

[36] Guy W (1976). ECDEU assessment manual for psychopharmacology.

[37] American Psychiatric Association (1994). Diagnostic and statistical manual of mental disorders.

[38] Ravens-Sieberer U, Erhart M, Rajmil L, Herdman M, Auquier P, the European KIDSCREEN Group (2010). Reliability, construct and criterion validity of the KIDSCREEN-10 score: a short measure for children and adolescents’ well-being and health-related quality of life. Qual Life Res.

[39] Kaess M, Parzer P, Mattern M, Resch F, Bifulco A, Brunner R (2011). Childhood Experiences of Care and Abuse (CECA): Validierung der deutschen Version von Fragebogen und korrespondierendem Interview sowie Ergebnisse einer Untersuchung von Zusammenhängen belastender Kindheitserlebnisse mit suizidalen Verhaltensweisen. Z Für Kinder.

[40] Pukrop R, Gentil I, Steinbring I, Steinmeyer E (2001). Factorial structure of the German version of the dimensional assessment of personality pathology-basic questionnaire in clinical and nonclinical samples. J Personal Disord.

[41] StataCorp (2019). Stata Statistical Software: Release 16.

[42] Vermunt JK, Magidson J (2016). Technical guide for latent GOLD 5.1: basic, advanced, and. Syntax..

[43] McLachlan GJ (1987). On bootstrapping the likelihood ratio test Stastistic for the number of components in a Normal mixture. J R Stat Soc Ser C Appl Stat.

[44] Schwarz G (1978). Estimating the dimension of a model. Ann Stat.

[45] Sclove SL (1987). Application of model-selection criteria to some problems in multivariate analysis. Psychometrika..

[46] Dziak JJ, Coffman DL, Lanza ST, Li R, Jermiin LS. Sensitivity and Specificity of Information Criteria [Internet]. Bioinformatics; 2018 Oct [cited 2019 Jul 25]. Available from: http://biorxiv.org/lookup/doi/10.1101/449751 10.1093/bib/bbz016 PMC7299313 30895308

[47] Nylund KL, Asparouhov T, Muthén BO (2007). Deciding on the number of classes in latent class analysis and growth mixture modeling: a Monte Carlo simulation study. Struct Equ Model Multidiscip J.

[48] Lubke GH, Neale M (2008). Distinguishing between latent classes and continuous factors with categorical outcomes: class invariance of parameters of factor mixture models. Multivar Behav Res.

[49] Raftery AE (1995). Bayesian model selection in social research. Sociol Methodol.

[50] Celeux G, Soromenho G (1996). An entropy criterion for assessing the number of clusters in a mixture model. J Classif.

[51] Nylund-Gibson K, Masyn KE (2016). Covariates and mixture modeling: results of a simulation study exploring the impact of Misspecified effects on class enumeration. Struct Equ Model Multidiscip J.

[52] McMahon K, Hoertel N, Peyre H, Blanco C, Fang C, Limosin F (2019). Age differences in DSM-IV borderline personality disorder symptom expression: results from a national study using item response theory (IRT). J Psychiatr Res.

[53] Schulte Holthausen B, Habel U (2018). Sex differences in personality disorders. Curr Psychiatry Rep..

[54] Benjamini Y, Hochberg Y (1995). Controlling the false discovery rate: a practical and powerful Appraoch to multiple testing. J R Stat Soc Ser B Methodol.

[55] World Health Organization (1992). The ICD-10 classification of mental and behavioural disorders: clinical descriptions and diagnostic guidelines.

[56] Chanen AM, Kaess M (2012). Developmental pathways to borderline personality disorder. Curr Psychiatry Rep.

[57] Steinmeyer EM, Klosterkötter J, Möller HJ, Sass H, Herpertz S, Czernik A (2002). Personality and personality disorders I. universality and sensitivity of dimensional personality models as diagnostic systems for personality disorders. Fortschr Neurol Psychiatr.

[58] Loeber R, Burke JD, Lahey BB (2002). What are adolescent antecedents to antisocial personality disorder?. Crim Behav Ment Health.

[59] Beauchaine TP, Klein DN, Crowell SE, Derbidge C, Gatzke-Kopp L (2009). Multifinality in the development of personality disorders: a biology × sex × environment interaction model of antisocial and borderline traits. Dev Psychopathol.

[60] Millon T, Millon C, Davis RD (1993). Millon adolescent clinical inventory.

[61] Westen D, Muderrisoglu S (2003). Assessing personality disorders using a systematic clinical interview: evaluation of an alternative to structured interviews. J Personal Disord.

[62] Eaton NR, Krueger RF, Keyes KM, Skodol AE, Markon KE, Grant BF (2011). Borderline personality disorder co-morbidity: relationship to the internalizing–externalizing structure of common mental disorders. Psychol Med.

[63] Krueger RF (1999). The structure of common mental disorders. Arch Gen Psychiatry.

[64] Bradley R, Zittel Conklin C, Westen D (2005). The borderline personality diagnosis in adolescents: gender differences and subtypes. J Child Psychol Psychiatry.

[65] Ramos V, Canta G, de Castro F, Leal I (2014). Discrete subgroups of adolescents diagnosed with borderline personality disorder: a latent class analysis of personality features. J Personal Disord.

[66] Poreh AM, Rawlings D, Claridge G, Freeman JL, Faulkner C, Shelton C (2006). The BPQ: A scale for the assessment of borderline personality based on DSM-IV criteria. J Personal Disord.

[67] Thompson KN, Jackson H, Cavelti M, Betts J, McCutcheon L, Jovev M (2019). The clinical significance of subthreshold borderline personality disorder features in outpatient youth. J Personal Disord.

[68] Kaess M, Fischer-Waldschmidt G, Resch F, Koenig J (2017). Health related quality of life and psychopathological distress in risk taking and self-harming adolescents with full-syndrome, subthreshold and without borderline personality disorder: rethinking the clinical cut-off?Borderline Personal. Disord Emot Dysregulation.

[69] Buerger A, Fischer-Waldschmidt G, Hammerle F, von Auer AK, Parzer P, Kaess M (2019). Differential change of borderline personality disorder traits during dialectical behavior therapy for adolescents. J Personal Disord.

[70] Kaess M, Edinger A, Fischer-Waldschmidt G, Parzer P, Brunner R, Resch F. Effectiveness of a brief psychotherapeutic intervention compared with treatment as usual for adolescent nonsuicidal self-injury: a single-centre, randomised controlled trial. Eur Child Adolesc Psychiatry [Internet]. 2019 11 [cited 2020 Jan 6]; Available from: http://link.springer.com/10.1007/s00787-019-01399-1 10.1007/s00787-019-01399-1 PMC7305262 31512050

[71] Lenzenweger MF, Clarkin JF, Yeomans FE, Levy KN, Kernberg OF (2008). Refining the borderline personality disorder phenotype through finite mixture modeling: implications for classification. J Personal Disord.

