# Supplementary material for: Heterogeneity of borderline personality disorder symptoms in help-seeking adolescents
Source: Borderline Personal Disord Emot Dysregul. 2021 Feb 26;8:9. doi: 10.1186/s40479-021-00147-9 (PMC7958409; doi:10.1186/s40479-021-00147-9)
Supplement: Supplementary file 1 — Additional file 1: Table S1. Specification and graphic illustration of FMM variants according to Clark et al. (2013). Figure S1. Graphical illustration of the best fitting two-class/one-factor FMM-3 model with different covariate effects [file 40479_2021_147_MOESM1_ESM.docx]

**Supplementary Material (SM) for:**

**Heterogeneity of Borderline Personality Disorder Symptoms**

**in Help-Seeking Adolescents**

This includes:

- SM Table 1
- SM Figure 1

***SM Table 1***. Specification and graphic illustration of FMM variants according to Clark et al. (2013)

| Parameters | FMM variants | | | |
| --- | --- | --- | --- | --- |
|  | FMM-1 | FMM-2 | FMM-3 | FMM-4 |
| Factor mean(s) | Class-variant | Class-variant | Fixed to zero | Fixed to zero |
| Factor variance(s) and covariance(s) | Fixed to zero | Class-variant | Class-variant | Class-variant |
| Item thresholds | Class-invariant | Class-invariant | Class-variant | Class-variant |
| Factor loadings | Class-invariant | Class-invariant | Class-invariant | Class-variant |
| Illustration | 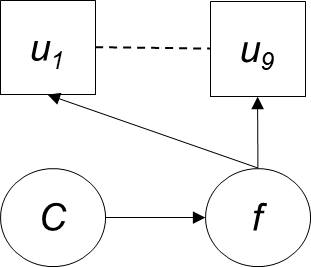 | 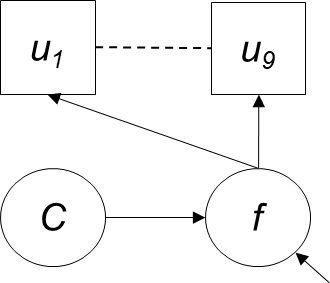 | 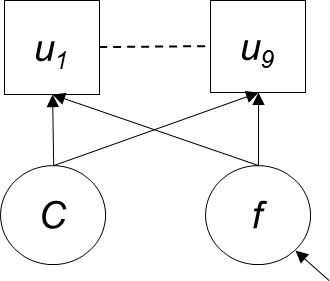 | 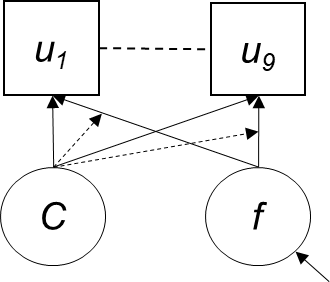 |
| Interpretation | There is no within-class heterogeneity. Class membership is based on each indivdiual’s location on the factor. The factor is measured the same way across classes. | There is within-class heterogeneity. Class membership is based on each indivdiual’s location on the factor. The factor is measured the same way across classes. | There are different amounts of heterogeneity within each class. Classes are based on the responses to the items rather than the factor means and variances. | There are different amounts of heterogeneity within each class. Classes are based on the responses to the items rather than the factor means and variances. The factor is not measured the same way across classes, indicating that there is a different factor within each class. |

*Notes. u* = DSM BPD criteria (observed variables), *C* = latent class variable, *f* = latent factor. Rectangles indicate observed variables. Circles indicate latent variables. The arrow pointing into the latent factor indicates a residual..

***SM Figure 1.*** Graphical illustration of the best fitting two-class/one-factor FMM-3 model with different covariate effects

***
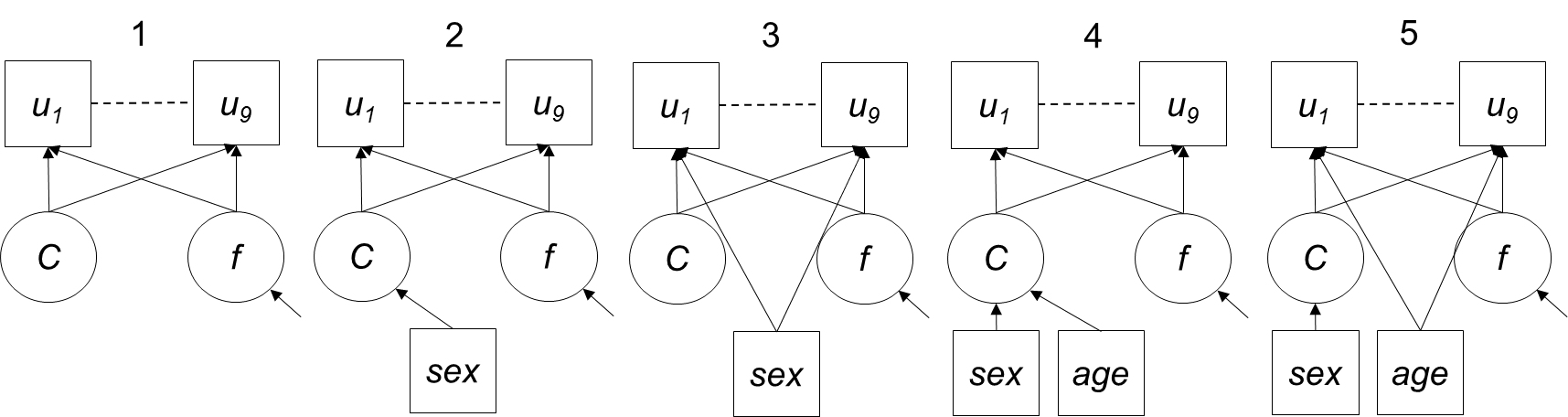
***

*Notes.* The model numbers correspond to the model numbers in Table 3. Rectangles indicate observed variables. Circles indicate latent variables. The arrow pointing into the latent factor indicates a residual.
